# A Ferroptosis-Related lncRNAs Signature Predicts Prognosis and Immune Microenvironment for Breast Cancer

**DOI:** 10.3389/fmolb.2021.678877

**Published:** 2021-06-07

**Authors:** Kaiming Zhang, Liqin Ping, Tian Du, Gehao Liang, Yun Huang, Zhiling Li, Rong Deng, Jun Tang

**Affiliations:** ^1^ Department of Breast Oncology, State Key Laboratory of Oncology in South China, Collaborative Innovation Center for Cancer Medicine, Sun Yat-Sen University Cancer Center, Guangzhou, China; ^2^ Department of Medical Oncology, State Key Laboratory of On cology in South China, Collaborative Innovation Center for Cancer Medicine, Sun Yat-Sen University Cancer Center, Guangzhou, China; ^3^ Department of Experimental Research, State Key Laboratory of Oncology in South China, Collaborative Innovation Center for Cancer Medicine, Sun Yat-Sen University Cancer Center, Guangzhou, China

**Keywords:** breast cancer, ferroptosis, lncRNA, prognostic signature, tumor immune microenvironment

## Abstract

**Background:** Ferroptosis, a regulated cell death which is driven by the iron-dependent peroxidation of lipids, plays an important role in cancer. However, studies about ferroptosis-related Long non-coding RNAs (lncRNAs) in breast cancer (BC) are limited. Besides, the prognostic role of ferroptosis-related lncRNAs and their relationship to immune microenvironment in breast cancer remain unclear. This study aimed to explore the potential prognostic value of ferroptosis-related lncRNAs and their relationship to immune microenvironment in breast cancer.

**Methods:** RNA-sequencing data of female breast cancer patients were downloaded from TCGA database. 937 patients were randomly separated into training or validation cohort in 2:1 ratio. Ferroptosis-related lncRNAs were screened by Pearson correlation analysis with 239 reported ferroptosis-related genes. A ferroptosis-related lncRNAs signature was constructed with univariate and multivariate Cox regression analyses in the training cohort, and its prognostic value was further tested in the validation cohort.

**Results:** An 8-ferroptosis-related-lncRNAs signature was developed by multivariate Cox regression analysis to divide patients into two risk groups. Patients in the high-risk group had worse prognosis than patients in the low-risk group. Multivariate Cox regression analysis showed the risk score was an independent prognostic indicator. Receiver operating characteristic curve (ROC) analysis proved the predictive accuracy of the signature. The area under time-dependent ROC curve (AUC) reached 0.853 at 1 year, 0.802 at 2 years, 0.740 at 5 years in the training cohort and 0.791 at 1 year, 0.778 at 2 years, 0.722 at 5 years in the validation cohort. Further analysis demonstrated that immune-related pathways were significantly enriched in the high-risk group. Analysis of the immune cell infiltration landscape showed that breast cancer in the high-risk group tended be immunologically “cold”.

**Conclusion:** We identified a novel ferroptosis-related lncRNA signature which could precisely predict the prognosis of breast cancer patients. Ferroptosis-related lncRNAs may have a potential role in the process of anti-tumor immunity and serve as therapeutic targets for breast cancer.

## Introduction

Breast cancer is the leading cause of female death worldwide (Bray et al., 2018). With the development in the diagnosis and treatment for breast cancer (Pondé et al., 2019; Ruddy and Ganz, 2019), the survival rate of breast cancer patients has been significantly improved, up to 90% of 5-year survival rate (DeSantis et al., 2019). However, as a heterogeneous cancer, breast cancer with the same TNM stage and immunohistochemical subtype may have totally different prognosis (Masood, 2005; Yersal and Barutca, 2014). It is necessary to find novel prognostic factors and therapeutic targets for breast cancer to guide clinical practice.

Ferroptosis is a new form of regulated cell death which is driven by the iron-dependent peroxidation of lipids (Dixon et al., 2012; Stockwell et al., 2017). Recent studies have found that ferroptosis is closely related to the tumorigenesis and progression of cancers, and activating the ferroptosis process of cancer cells is a novel strategy to treat cancers, especially for routine therapy-resistant cancers (Hassannia et al., 2019; Mou et al., 2019). It has been proved that resistance to ferroptosis reduced the efficacy of sorafenib and was associated with poor prognosis in hepatocellular carcinoma (Houessinon et al., 2016; Liang et al., 2020). Lu et al. reported that ferroptosis was suppressed in colorectal cancer, leading to short overall survival of colorectal cancer patients (Lu et al., 2020). Similarly, ferroptosis plays an important role in breast cancer. Recent research has found that prominin2 (PROM2) could reduce the ferroptosis of breast cancer cells and promote tumor progression by stimulating iron export (Brown et al., 2019). Additionally, another study has reported that GSK3β/Nrf2 signaling pathway was activated in breast cancer, increasing the expression level of Nrf2 to resist ferroptosis (Wu et al., 2020). These studies suggest that resistance of ferroptosis is important for the progression of breast cancer. Thus, we may be able to treat breast cancer by inducing ferroptosis. Ma et al. found that Siramesine and Lapatinib could induce ferroptosis in breast cancer cells and could be a potential therapeutic regimen for breast cancer patients (Ma et al., 2016). Therefore, it is necessary to identify more biomarkers associated with ferroptosis for breast cancer. However, the current studies about relationship between breast cancer and ferroptosis are limited, the association between ferroptosis and prognosis of breast cancer patients remains unclear. Therefore, it is meaningful to identify novel ferroptosis-related biomarkers not only for elucidating the specific mechanism of ferroptosis in breast cancer but also for predicting prognosis of breast cancer patients.

The long non-coding RNAs (lncRNAs) are defined as a class of RNAs with more than 200 nucleotides in length and without protein-coding ability (Guttman and Rinn, 2012). Although lncRNAs could not code protein in cells, they still have several specific functions, such as regulation of transcription, regulation of mRNA processing and regulation of mRNA post transcriptional control (Beermann et al., 2016). A recent study has found that lncRNA has a role in regulating ferroptosis in breast cancer (Mao et al., 2018). However, there are few studies focusing on the ferroptosis-related-lncRNAs in breast cancer, and a large number of lncRNAs regulating ferroptosis have not been found. The development of high-throughput sequencing technology is helpful to identify potential ferroptosis-related biomarkers and prognostic biomarkers (Desany and Zhang, 2004; Huang et al., 2009).

In recent years, immune checkpoint blockade (ICB) therapy has made great breakthroughs in immunotherapy for cancer patients (Garon et al., 2015). The response to ICB therapy is closely related to the tumor microenvironment. In tumors with less infiltrating cytotoxic T cells and lower PD-L1 expression, which are defined as immune “cold” tumors, the efficacy of ICB therapy is often poor (Chen et al., 2017). Unfortunately, most breast cancers are “cold” tumors (Tekpli et al., 2019). Transforming “cold” tumors into “hot” tumors may enhance the response of breast cancer to ICB therapy (Song et al., 2020). Currently, studies about the relationship between ferroptosis and anti-tumor immunity are few. Wang et al. discovered that the anti-tumor function of CD8^+^ T cells was in part mediated by promoting tumor ferroptosis (Wang et al., 2019b). Exploring the relationship between ferroptosis and tumor immune microenvironment can help us better understand the pathogenesis of “cold” breast cancer and provide potential treatment strategies for “cold” breast cancer.

In this study, we aimed to recognize ferroptosis-related lncRNAs in breast cancer, which can not only provide important insight on the molecular and signaling pathways of ferroptosis in breast cancer, but also be used to predict the prognosis of breast cancer patients. Furthermore, we found the relationship between ferroptosis and the immune microenvironment in breast cancer, providing a theoretical reference for the treatment strategies of “cold” breast cancer.

## Materials and Methods

### Patient Data Acquisition

**FIGURE 1:**
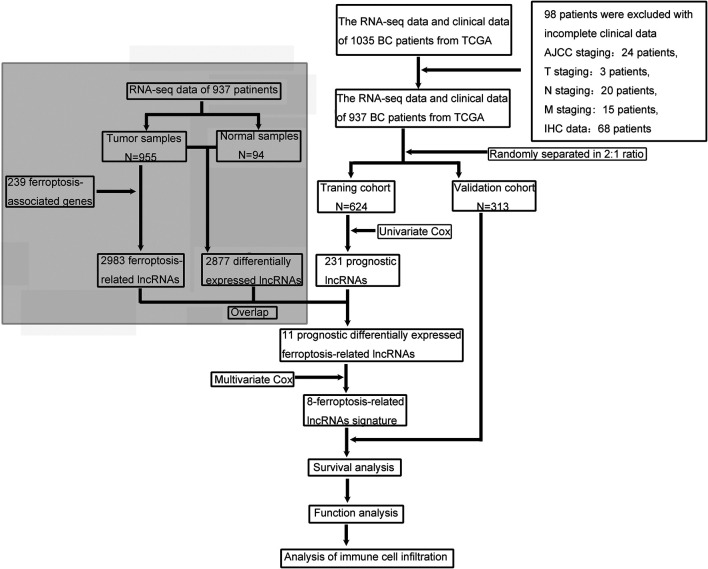
The flow chart of our study.

The data of RNA-sequencing (RNA-seq) and corresponding clinical information of 1,035 female patients with ductal or lobular breast cancer were downloaded from TCGA database (https://portal.gdc.cancer.gov/repository). lncRNAs and protein-coding genes were classified by using the ensembl human genome browser GRCh38. p13 (http://asia.ensembl.org/index.html) (Cunningham et al., 2019). Patients were excluded if the information of clinical characteristics were incomplete, and AJCC staging data were not available for 24 patients, T staging data for 3 patients, N staging data for 20 patients, M staging data for 15 patients and IHC subtype data for 68 patients. Patients with one or more unavailable clinical characteristics were excluded. Eventually, a total of 98 patients were excluded from the study (Figure 1). The data from TCGA is publicly available, and the current research followed the TCGA data access policies and publication guidelines.

### Identification of Ferroptosis-Related lncRNAs

239 ferroptosis-associated genes were identified from the ferroptosis database (FerrDb; http://www.zhounan.org/ferrdb/operations/download.html), which contained the most exhaustive list of ferroptosis-related genes (Zhou and Bao, 2020). Then the Pearson correlation coefficients were calculated to define the correlation between the expression of ferroptosis-related genes and corresponding lncRNAs. The ferroptosis-related lncRNAs were identified according to the standard that the *p* value was less than 0.001 (*p* < 0.001) and the absolute value of Pearson correlation coefficient was more than 0.3 (|R| > 0.3).

### Construction and Validation of a Prognostic Ferroptosis-Related lncRNAs Signature.

The “edgeR” R package was used to screen the differentially expressed lncRNAs between tumor tissues and nontumorous tissues with a false discovery rate (FDR) < 0.05 and |log2FC| ≥ 1. A total of 937 patients were randomly separated into training or validation cohort in 2:1 ratio for constructing and validating the ferroptosis-related lncRNAs signature. Univariate Cox regression analysis of overall survival (OS) was performed to identify prognostic lncRNAs which were associated (*p* < 0.05) with the OS in the training cohort. The common lncRNAs of differentially expressed lncRNAs, ferroptosis-related lncRNAs and prognostic lncRNAs were identified as the candidate lncRNAs for the ferroptosis-related lncRNAs prognostic signature. Then, the candidate ferroptosis-related lncRNAs underwent multivariate Cox regression analysis to evaluate their contribution as prognostic factors in overall survival of patients. Finally, we identified 8 optimal ferroptosis-related lncRNAs for the prognostic model, which was constructed based on lowest Akaike information criterion (AIC) value. The risk score of every patient based on this prognostic signature was calculated according to the normalized expression level of ferroptosis-related lncRNAs and corresponding regression coefficients. The computational formula was as follows:

Risk Score = e^sum (each lncRNA’s expression × corresponding regression coefficient)^. The patients in the training cohort were divided into low-risk and high-risk groups according to the median value of the risk score and the overall survival between different groups were compared by Kaplan-Meier analysis with the log-rank test. Then, time-dependent ROC curve analysis was performed with “survivalROC” R package to evaluate the predictive accuracy of the ferroptosis-related lncRNAs signature. To validate this prognostic model, the risk score for each patient in the validation cohort was calculate based on the same formula as the training cohort, and patients in validation cohort were divided into low-risk and high-risk groups according to the same cut-off value as the training cohort, then the Kaplan-Meier analysis and the time-dependent ROC curve analyses were performed.

### Construction of the lncRNA-mRNA Co-Expression Network

In order to demonstrate the correlation between the ferroptosis-related lncRNAs and their corresponding mRNAs, the mRNA-lncRNA co-expression network was constructed and visualized using the Cytoscape software (version 3.7.2, http://www.cytoscape.org/). Then, the Sankey diagram was plotted to further demonstrate the degree of correlation between ferroptosis-related lncRNAs(risk/protective) and their corresponding mRNAs.

### Gene Set Enrichment Analysis

The “edgeR” R package was used to identify the differentially expressed genes between the high-risk and low-risk groups with a false discovery rate (FDR) < 0.05 and |log2FC| ≥ 1. *p* values were adjusted with the Benjamini and Hochberg (BH) method. And the differentially expressed genes underwent gene set enrichment analysis (GSEA; http://www.broadinstitute.org/gsea) to explore the molecular and biological differences between tumors of patients in different groups. The gene sets were filtered using the minimum and maximum gene set size of 10 and 500 genes, respectively.

### Functional Enrichment Analysis

The differentially expressed genes between high-risk and low-risk groups underwent Gene Ontology (GO) analysis to determine the biological processes, molecular functions, and cellular components related to the ferroptosis-related lncRNAs signature. And the Kyoto Encyclopedia of Genes and Genomes (KEGG) pathway analysis was performed to identify the signaling pathways associated with the ferroptosis-related lncRNAs signature.

### Estimation of Tumor-Infiltrating Immune Cells

The proportion of different tumor-infiltrating immune cells were calculated by CIBERSORT algorithm (Newman et al., 2015). The normalized gene expression data was uploaded to the CIBERSORT web portal (http://cibersort.stanford.edu/), and the algorithm was based on LM22 gene signature and 1,000 permutations. The samples were filtered based on *p* value <0.05. The results produced by CIBERSORT were analyzed subsequently.

### Statistical Analysis

Wilcox-test was used to compare proportion of tumor-infiltrating immune cells and expression levels of immune checkpoint molecules between high-risk and low-risk groups. Spearman correlation analysis was used to analyze the correlation between tumor-infiltrating immune cells. Differences in proportions of clinical characteristics were analyzed by the Chi-squared test. Univariate Cox regression analysis and multivariate Cox regression analysis were performed to identify independent prognostic factors of OS. The predictive accuracy of the prognostic model for OS was evaluated by performing time-dependent ROC curve analysis. All statistical analyses were conducted by SPSS (Version 23.0) or R software (Version 3.5.3). Statistical significance was defined as *p* value <0.05, and all *p* values were two-tailed.

## Results

### The Clinical Characteristics of Patients in Training Cohort and Validation Cohort Were Consistent

A total of 937 female breast cancer patients were randomly separated into training (*n* = 624) or validation (*n* = 313) cohorts in 2:1 ratio. Table 1 presented the detailed clinical characteristics of these patients. There was no significant difference between the clinical characteristics of patients in the training cohort and the validation cohort.

**TABLE 1:** Clinical characteristics of patients in training cohort and validation cohort.

Variables	Training cohort (*n* = 624)		Validation cohort (*n* = 313)		*p*-value
	NO.	%	NO.	%	
Age	—	—	—	—	—
Median (years)	58.5	—	57	—	—
≤60	341	54.6	180	57.5	0.406
>60	283	45.4	133	42.5	—
AJCC Stage	—	—	—	—	0.820
I	107	17.2	52	16.7	—
II	59	57.5	187	59.7	—
III	149	23.9	68	21.7	—
IV	9	1.4	6	1.9	—
T Stage	—	—	—	—	0.979
T1	160	25.6	81	25.9	—
T2	366	58.7	186	59.4	—
T3	82	13.1	38	12.1	—
T4	16	2.6	8	2.6	—
N stage	—	—	—	—	0.732
N0	290	46.5	154	49.2	—
N1	218	34.9	99	31.6	—
N2	69	11.1	38	12.1	—
N3	47	7.5	22	7.0	—
M Stage	—	—	—	—	0.585
M0	615	98.6	307	98.1	—
M1	9	1.4	6	1.9	—
IHC Subtype	—	—	—	—	0.859
Luminal	501	80.3	256	81.8	—
HR−, Her2+	24	3.8	11	3.5	—
Triple negative	99	15.9	46	14.7	—

### Identification of Prognostic Differentially Expressed Ferroptosis-Related lncRNAs in Breast Cancer Patients

**FIGURE 2:**
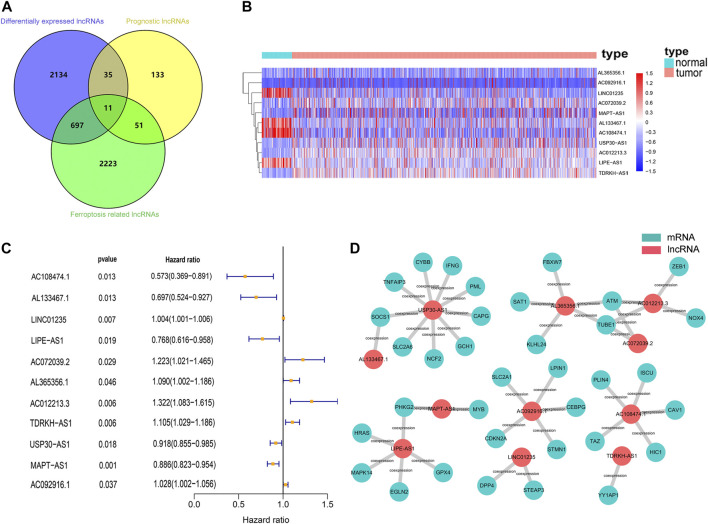
Identification of prognostic differentially expressed ferroptosis-related lncRNAs in breast cancer patient. **(A)** Venn diagram to identify the common lncRNAs of differentially expressed lncRNAs, ferroptosis-related lncRNAs and prognostic lncRNAs. **(B)** The 11 overlapping lncRNAs were differently expressed in normal and tumor tissue. **(C)** Forest plots showing the results of the univariate Cox regression analysis between overlapping lncRNAs and overall survival. **(D)** lncRNA-mRNA co-expression network of candidate lncRNAs and ferroptosis-related genes.

Firstly, 13,683 lncRNAs were identified by analyzing the RNA-seq data of the breast cancer patients, among which 2,877 lncRNAs were differentially expressed between normal and tumor tissue. And a total of 231 prognostic lncRNAs were identified by univariate Cox regression analysis in the training cohort. To identify ferroptosis-related lncRNAs, we downloaded 239 ferroptosis-associated genes from the ferroptosis database (Zhou and Bao, 2020). The expression of 2,983 ferroptosis-related lncRNAs were found to be correlated (|R| > 0.3 and *p* < 0.001) with the expression of ferroptosis-related genes. Finally, we found 11 lncRNAs (AC108474.1, AL133467.1, LINC01235, AC072039.2, AL365356.1, AC012213.3, TDRKH-AS1, USP30-AS1, MAPT-AS1, AC092916.1, and LIPE-AS1) that were present in all three sets of differentially expressed lncRNAs, prognostic lncRNAs and ferroptosis-related lncRNAs (Figure 2A). These 11 lncRNAs were not only differentially expressed lncRNAs between normal and tumor tissue but also prognostic lncRNAs related to the OS and ferroptosis-related lncRNAs (Figures 2B–D).

### Construction of a Prognostic Model in the Training Cohort

**FIGURE 3:**
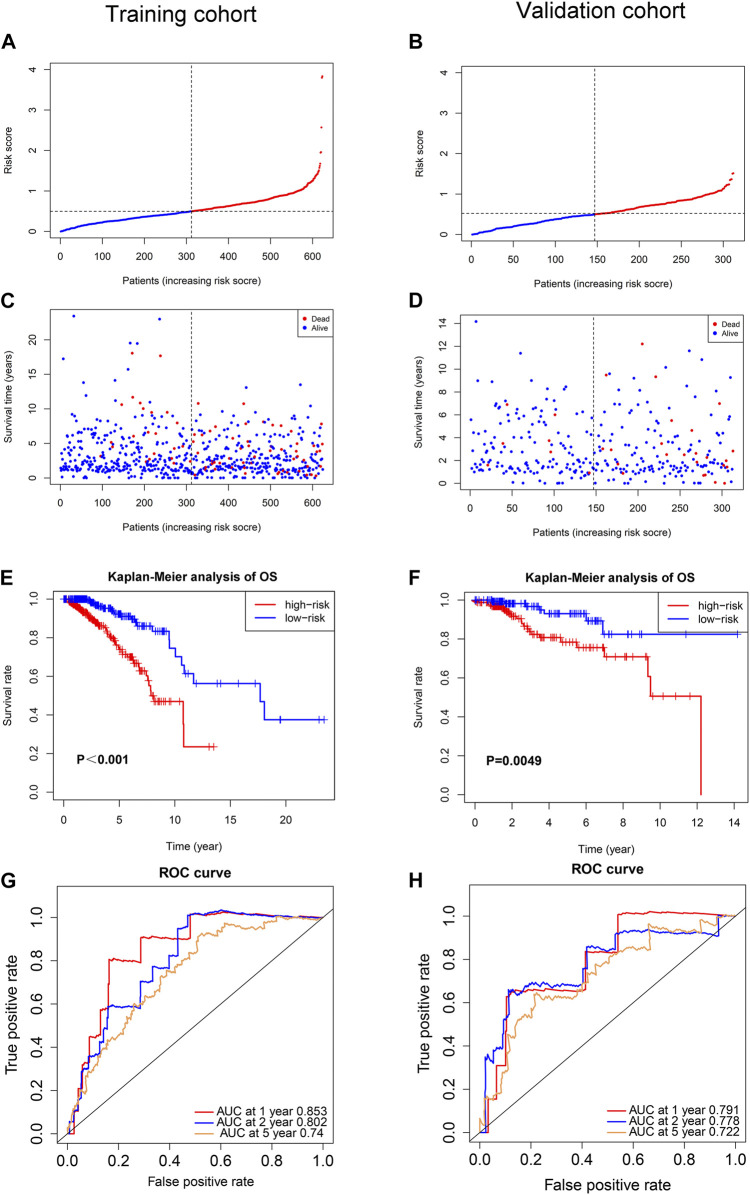
Prognostic analysis of the ferroptosis-related lncRNAs signature model in the training cohort and validation cohort. **(A)** The distribution of the risk scores in the training cohort. **(B)** The distribution of the risk scores in the validation cohort. **(C)** The distributions of overall survival status, overall survival and risk score in the training cohort. **(D)** The distributions of overall survival status, overall survival and risk score in the validation cohort. **(E)** Kaplan-Meier curves for the overall survival of patients in the high- and low-risk groups in the training cohort. **(F)** Kaplan-Meier curves for the overall survival of patients in the high- and low-risk groups in the validation cohort. **(G)** AUC of time-dependent ROC curves verified the prognostic accuracy of the risk score in the training cohort. **(H)** AUC of time-dependent ROC curves verified the prognostic accuracy of the risk score in the validation cohort.

Multivariate Cox regression analysis was performed in the training cohort to construct a prognostic model for OS using the expression level of the 11 ferroptosis-related lncRNAs. An optimal 8-lncRNAs (AC108474.1, AL133467.1, LINC01235, AC072039.2, TDRKH-AS1, USP30-AS1, MAPT-AS1, and LIPE-AS1) signature was identified based on the lowest Akaike information criterion (AIC) (Table 2). The risk score was calculated with the following formula: e^(−0.325 × expression level of AC108474.1−0.270 × expression level of AL133467.1 + 0.002 × expression level of LINC01235−0.171 × expression level of LIPE-AS1 + 0.195 × expression level of AC072039.2 + 0.086 × expression level of TDRKH-AS1-0.074 × expression level of USP30−AS1−0.132 × expression level of MAPT-AS1)^. The patients were further divided into high-risk group (*n* = 312) and low-risk group (*n* = 312) by the median risk score as cut-off value. The risk score was significantly associated with AJCC stage, N stage and IHC subtype of breast cancer patients (Table 3). As shown in Figures 3A, C, patients in the high-risk group had a higher probability of death than patients in the low-risk group. And the Kaplan-Meier analysis showed that patients in the high-risk group had significantly worse overall survival (OS) than patients in the low-risk group (Figure 3E, *p* < 0.001). As shown in Figure 3G, the area under ROC curve (AUC) reached 0.853 at 1 year, 0.802 at 2 years, and 0.740 at 5 years. Then, disease specific survival (DSS) and progression-free interval (PFI) were also analyzed to evaluate the effect of ferroptosis-related lncRNAs on prognosis, the results showed that patients in the high-risk group had shorter DSS and PFI than patients in the low-risk group (Supplemental Figures S1A,C).

**TABLE 2:** Akaike information criterion for the prognostic signature.

Model	Prognostic signature combination	AIC
1	AC108474.1 + AL133467.1 + LINC01235 + AC072039.2 + AL365356.1 + AC012213.3 + TDRKH-AS1 + USP30-AS1 + MAPT-AS1 + AC092916.1 + LIPE-AS1	1,166
2	AC108474.1 + AL133467.1 + LINC01235 + AC072039.2 + AC012213.3 + TDRKH-AS1 + USP30-AS1 + MAPT-AS1 + AC092916.1 + LIPE-AS1	1,164
3	AC108474.1 + AL133467.1 + LINC01235 + AC072039.2 + AC012213.3 + TDRKH-AS1 + USP30-AS1 + MAPT-AS1 + LIPE-AS1	1,162.48
4	AC108474.1 + AL133467.1 + LINC01235 + AC072039.2 + TDRKH-AS1 + USP30-AS1 + MAPT-AS1 + LIPE-AS1	1,161.48

**TABLE 3:** Relationship between risk score and clinical characteristics of patients in the training cohort.

Variables	Low-risk score (*n* = 312)		High-risk score (*n* = 312)		*p*-value
	NO.	%	NO.	%	
Age	—	—	—	—	0.809
≤60	172	55.1	169	54.2	—
>60	140	44.9	143	45.8	—
AJCC Stage	—	—	—	—	0.020
I	66	21.1	41	13.2	—
II	179	57.4	180	57.7	—
III	64	20.5	85	27.2	—
IV	3	1.0	6	1.9	—
T Stage	—	—	—	—	0.058
T1	94	30.1	66	21.2	—
T2	171	54.8	195	62.5	—
T3	41	13.1	41	13.1	—
T4	6	1.9	10	3.2	—
N stage	—	—	—	—	0.028
N0	155	47.9	135	43.3	—
N1	110	35.3	108	34.6	—
N2	23	7.4	46	14.7	—
N3	24	7.7	23	7.4	—
M Stage	—	—	—	—	0.314
M0	309	99.0	306	98.1	—
M1	3	1	6	1.9	—
IHC Subtype	—	—	—	—	0.007
Luminal	262	84.0	239	76.6	—
HR-, Her2+	5	1.6	19	6.1	—
Triple negative	45	14.4	54	17.3	—

### Validation of the 8-Ferroptosis-related-lncRNAs Signature in the Validation Cohort

To test the reliability of the ferroptosis-related-lncRNAs signature constructed from the training cohort, risk scores were calculated for the patients in the validation cohort with the formula: e^(−0.325 × expression level of AC108474.1 −0.270 × expression level of AL133467.1 + 0.002 × expression level of LINC01235 −0.171 × expression level of LIPE-AS1 + 0.195 × expression level of AC072039.2 + 0.086 × expression level of TDRKH-AS1 −0.074 × expression level of USP30-AS1−0.132 × expression level of MAPT-AS1)^, which is the exact same formula as in the training cohort. Then the patients in validation cohort were divided into high-risk group (*n* = 166) and low-risk group (*n* = 147) by the same cut-off value as the training cohort. Similar to the training cohort, patients in the high-risk group of validation cohort had a higher probability of death than patients in the low-risk group (Figures 3B, D). And the Kaplan-Meier analysis showed that patients in the high-risk group had worse OS than patients in the low-risk group (Figure 3F, *p* = 0.0049). As shown in Figure 3H, the area under ROC curve (AUC) reached 0.791 at 1 year, 0.778 at 2 years, and 0.722 at 5 years. To further verify the effect of ferroptosis-related lncRNAs on prognosis, disease specific survival (DSS) and progression-free interval (PFI) were analyzed in the validation cohort, the results showed that high-risk patients had shorter DSS of and PFI than low-risk patients (Supplemental Figures S1B,D).

### Relationship Between the 8-Ferroptosis-related-lncRNAs Signature and Prognosis of Patients Receiving Different Treatment Strategies

**FIGURE 4:**
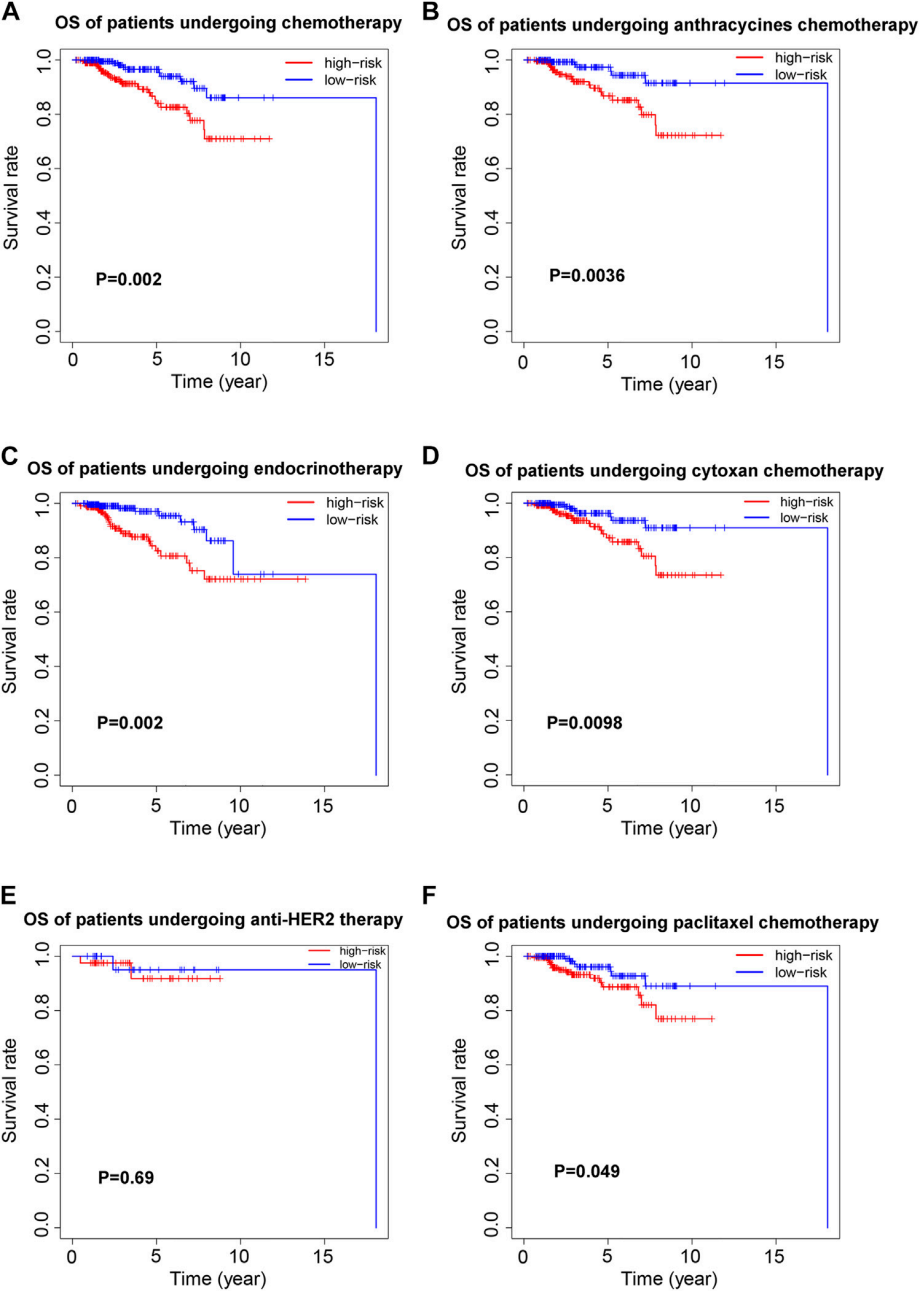
Prognostic value of the 8-ferroptosis-related-lncRNAs signature in patients receiving different treatment regimens. **(A)** Kaplan-Meier curves for the overall survival of high- and low-risk patients receiving chemotherapy. **(B)** Kaplan-Meier curves for the overall survival of high- and low-risk patients receiving anthracycline chemotherapy. **(C)** Kaplan-Meier curves for the overall survival of high- and low-risk patients receiving endocrinotherapy. **(D)** Kaplan-Meier curves for the overall survival of high- and low-risk patients receiving cyclophosphamide chemotherapy. **(E)** Kaplan-Meier curves for the overall survival of high- and low-risk patients receiving anti-HER2 therapy. **(F)** Kaplan-Meier curves for the overall survival of high- and low-risk patients receiving paclitaxel chemotherapy.

To investigate the prognostic value of the 8-ferroptosis-related-lncRNAs signature in patients receiving different treatment regimens, we collected and analyzed the treatment data of breast cancer patients from the TCGA database. First, we analyzed the prognostic role of ferroptosis-related lncRNAs in patients receiving chemotherapy, and the results showed that among all patients receiving chemotherapy, patients with high-risk score based on ferroptosis-related lncRNAs signature had worse prognosis (Figure 4A). We then analyzed the prognostic role of ferroptosis-related lncRNAs in patients receiving endocrinotherapy (Figure 4C), and we found that among all patients receiving endocrinotherapy, patients with high-risk score had worse prognosis. We also analyzed the prognostic role of ferroptosis-related lncRNAs in patients receiving anti-HER2 therapy, and the results showed no significant difference in prognosis between patients with high- and low-risk score (Figure 4E). To further explore the role of ferroptosis-related lncRNAs in different chemotherapeutic therapies, we analyzed the prognostic value of ferroptosis-related lncRNAs in patients treated with anthracycline, cyclophosphamide or paclitaxel, respectively. The results showed that high-risk patients had worse outcomes among patients treated with anthracycline, cyclophosphamide or paclitaxel (Figures 4B,D,F).

### Independent Prognostic Value of the 8-Ferroptosis-Related-lncRNAs Signature

**FIGURE 5:**
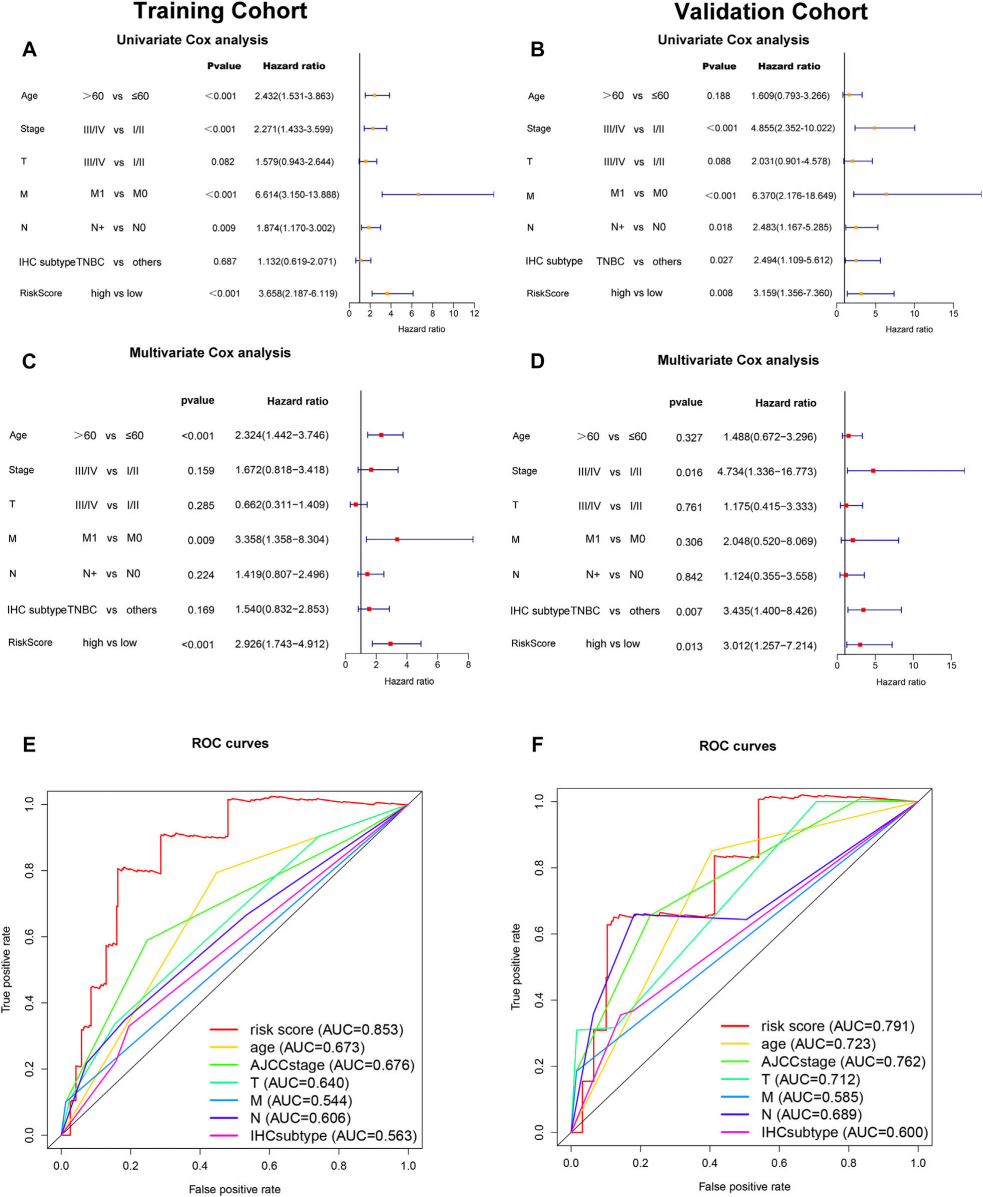
Independent prognostic value of the ferroptosis-related-lncRNAs signature. Results of the univariate Cox regression analysis and multivariate Cox regression analysis regarding OS in the training cohort **(A,C)** and the validation cohort **(B,D)**. AUC of ROC curves compared the prognostic accuracy of the risk score and other prognostic factors in the training cohort and validation cohort **(E,F)**.

To determine whether the risk score was an independent prognostic factor for breast cancer patients, univariate Cox regression analysis and multivariate Cox regression analysis were performed among the clinical characteristics and risk score. The results of univariate Cox regression analysis showed that the risk score was significantly associated with OS in both training cohort and validation cohort (Training cohort: HR = 3.658, 95% CI = 2.187–6.119, *p* < 0.001; Validation cohort: HR = 3.159, 95% CI 1.356–7.360, *p* = 0.008, respectively) (Figures 5A,C). After adjusting for other confounders, the risk score remained an independent predictor of OS in multivariate Cox regression analysis (Training cohort: HR = 2.926, 95% CI = 1.743–4.912, *p* < 0.001; Validation cohort: HR = 3.012, 95% CI = 1.257–7.214, *p* = 0.013; (Figures 5B,D). Furthermore, the ROC curve analysis showed that the AUC value of the ferroptosis-related lncRNAs prognostic signature in training cohort and validation cohort were 0.853 and 0.791, respectively, which were higher than the AUC values of other prognostic factors. (Figures 5E,F).

### Construction of the lncRNA–mRNA Co-Expression Network

**FIGURE 6:**
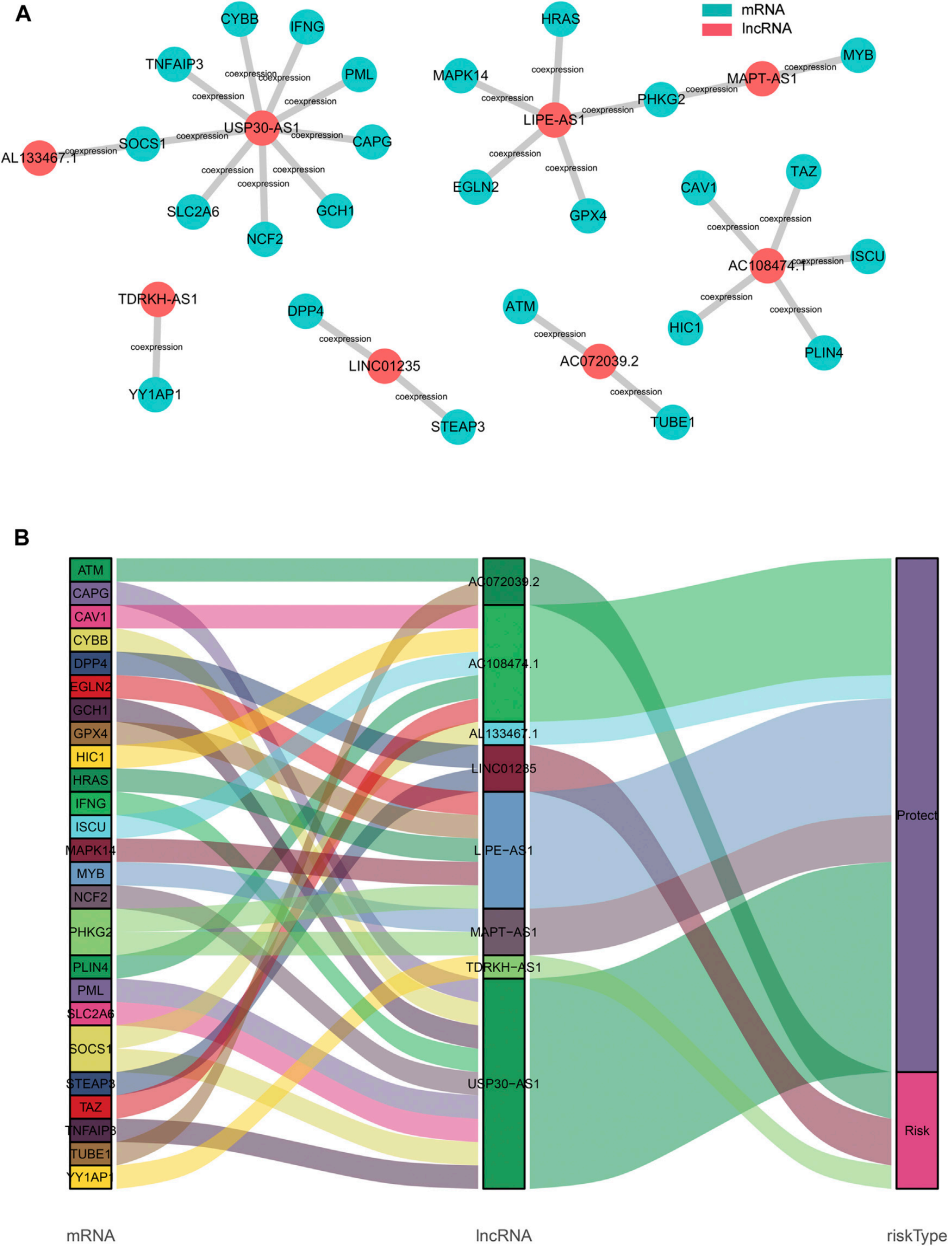
Construction of the ferroptosis-related lncRNA–mRNA co-expression network **(A)**. Diagram of the ferroptosis-related lncRNA–mRNA network **(B)**. The Sankey diagram shows the connection degree between the ferroptosis-related lncRNAs and ferroptosis-related genes.

To explore the potential roles of the 8 ferroptosis-related lncRNAs in breast cancer, the lncRNA-mRNA co-expression network was constructed using Cytoscape. The lncRNA-mRNA co-expression network contained 27 lncRNA-mRNA pairs (Figure 6A). Among these, LncRNA USP30−AS1 had co-expression relationship with 9 ferroptosis-related genes (CYBB, SOCS1, IFNG, TNFAIP3, PML, GCH1, NCF2, SLC2A6, and CAPG), LncRNA LIPE-AS1 was co-expressed with 5 ferroptosis-related genes (HRAS, PHKG2, MAPK14, EGLN2, and GPX4) and there was a co-expression relationship between AC108474.1 and 5 ferroptosis-related genes (TAZ, ISCU, CAV1, PLIN4, and HIC1). The Sankey diagram not only demonstrated the relationship between ferroptosis-related lncRNAs and ferroptosis-related genes, but also demonstrated the relationship between ferroptosis-related lncRNAs and OS of breast cancer patients (Figure 6B).

### Discovery of Important Pathways by Gene Set Enrichment Analysis

**FIGURE 7:**
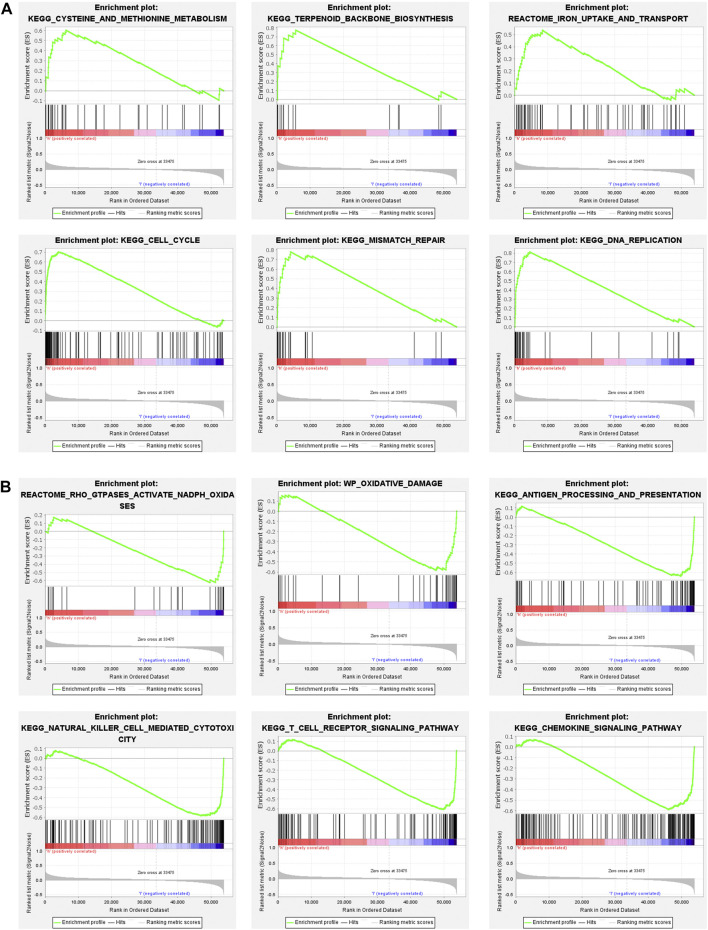
Gene set enrichment analysis (GSEA) of high-risk group and low-risk group based on the ferroptosis-related lncRNAs prognostic signature. **(A)** GSEA results show significant enrichment of antioxidant pathways and cell proliferation pathways in the high-risk breast cancer patients. **(B)** GSEA results show significant enrichment of oxidative damage-related pathways and immunoregulatory pathways in the low-risk breast cancer patients.

To explore the biological functions and signal transduction pathway associated with the ferroptosis-related lncRNAs, the differentially expressed genes between the high-risk and low-risk groups were used to perform Gene set enrichment analysis (GSEA). The results showed that genes in the antioxidant pathways and cell proliferation pathways, such as cysteine and methionine metabolism, terpenoid backbone biosynthesis, iron uptake and transport, cell cycle, DNA replication and mismatch repair were upregulated in the high-risk breast cancer patients (Figure 7A), while oxidative damage-related pathways were significantly down-regulated, such as GTPases activate NADPH oxidases and oxidative damage (Figure 7B). Interestingly, immunoregulatory pathways against cancer were significantly upregulated in the low-risk breast cancer patients, including pathways related to antigen processing and presentation, natural killer cell-mediated cytotoxicity, T cell receptor signaling and chemokine signaling (Figure 7B).

### Immune-Related Pathways Were Activated in the Low-Risk Group

**FIGURE 8:**
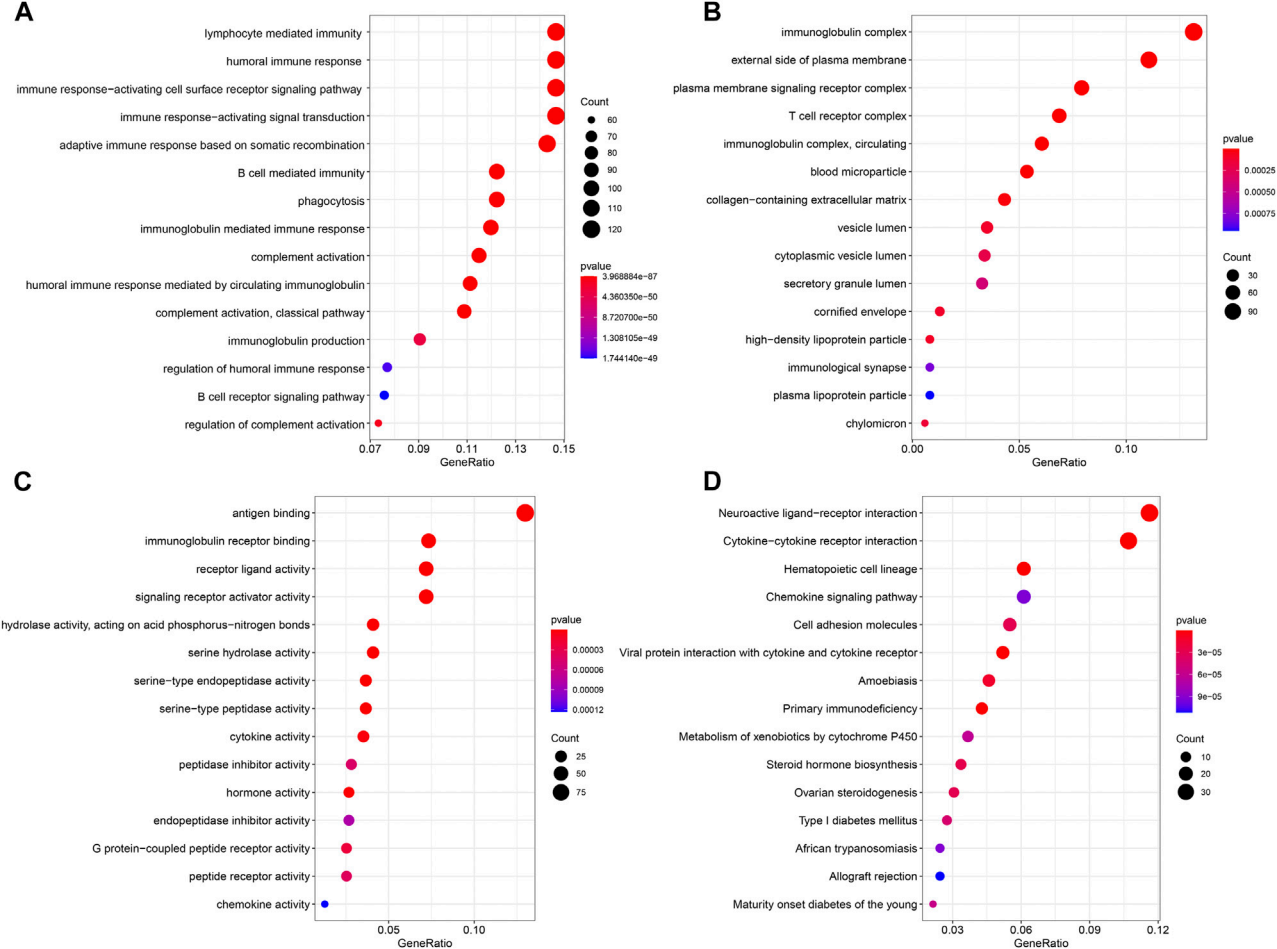
Results of GO and KEGG analyses. GO analysis showed differentially expressed genes between high- and low-risk groups were obviously enriched in immune-related biological processes **(A)**, immune-related cell components **(B)**, and immune-related molecular functions **(C)**. KEGG analysis showed differentially expressed genes were enriched in immune-related pathway **(D)**.

To further determine the biological functions related to the ferroptosis-related lncRNAs, the differentially expressed genes between the high-risk and low-risk groups were used to perform GO enrichment analysis and KEGG pathway analysis. We found that the differentially expressed genes were obviously enriched in many immune-related biological processes, such as lymphocyte mediated immunity, humoral immune response and immune response−activating cell surface receptor signaling pathway. (Figure 8A). The differentially expressed genes were also obviously enriched in many immune-related cellular components, such as immunoglobulin complex and T cell receptor complex (Figure 8B). In terms of molecular functions, the differentially expressed genes were enriched in many immune-related molecular functions, including antigen binding and immunoglobulin receptor binding (Figure 8C). The result of KEGG pathway analysis also showed that the differentially expressed genes were enriched in immune-related pathway, such as cytokine−cytokine receptor interaction, chemokine signaling pathway and primary immunodeficiency (Figure 8D).

### The Immune Cell Infiltration Landscape in Breast Cancer

**FIGURE 9:**
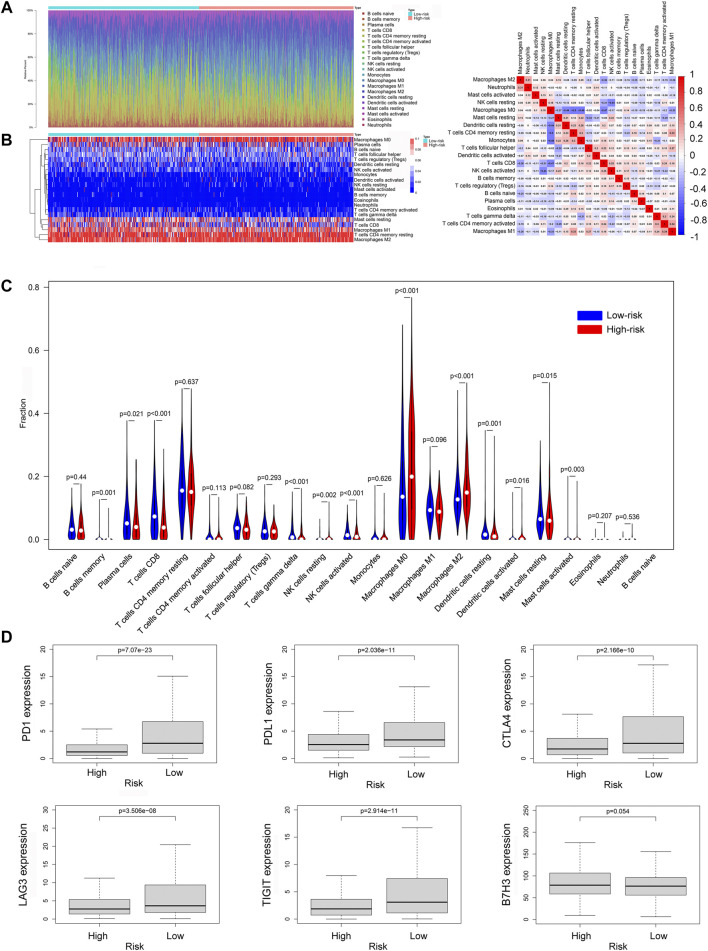
The immune cell infiltration landscape in breast cancer. **(A)** Barplot of the tumor-infiltrating cell proportions. **(B)** Heatmap of the tumor-infiltrating cell proportions. **(C)** Correlation matrix of immune cell proportions. **(D)** Violin plot showed the different proportions of tumor-infiltrating cells between high-risk group and low-risk group. **(E)** The expression levels of immune checkpoint molecules in high-risk group and low-risk group.

To further explore the relationship between ferroptosis-related lncRNAs and anti-tumor immunity, we screened the RNA-seq datasets of the 937 breast cancer patients using CIBERSORT algorithm, investigating the immune cell infiltration landscape. As shown in Figures 9A,B, the proportions of tumor-infiltrating immune cells between high-risk group and low-risk group were significantly different. And the correlation matrix of all tumor-infiltrating immune cell proportions was demonstrated in Figure 9C. To compare the differences of infiltrating immune cells between the high-risk and low-risk groups, we constructed a violin plot showing that the proportions of tumor-infiltrating CD8^+^ T cell, activated NK cell and M1 macrophages in high-risk group were significantly lower than those in low-risk group, while the proportion of tumor-infiltrating M2 macrophages in high-risk group was much higher than that in low-risk group (Figure 9D). Then we compared the expression levels of immune checkpoint molecules in high-risk group and low-risk group, the results showed that the expression levels of common immune checkpoint molecules such as PD1, PDL1, CTLA4, TIGIT, and LAG3 in low-risk group were all higher than those in high-risk group (Figure 9E).

### The Ferroptosis-Related lncRNAs Signature in Different Immunohistochemical Subtypes of Breast Cancer

**FIGURE 10:**
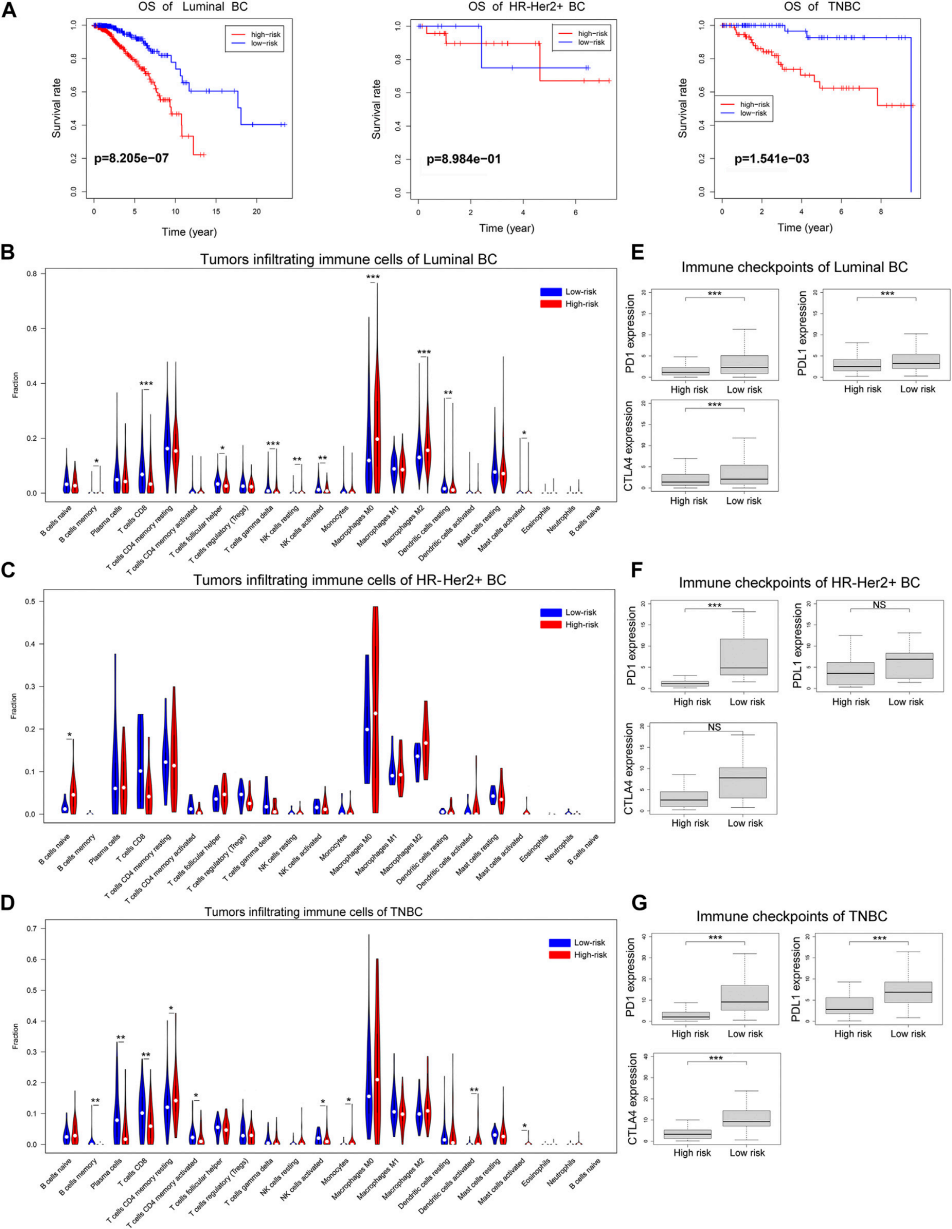
The overall survival and immune cell infiltration landscape in different immunohistochemical subtypes of breast cancer. **(A)** Kaplan-Meier curves for the overall survival of patients in the high- and low-risk groups in different immunohistochemical subtypes of breast cancer. **(B–D)** Violin plot showed the different proportions of tumor-infiltrating cells between high-risk group and low-risk group in luminal breast cancer, HR-Her2+ breast cancer or TNBC. **(E–G)** The expression levels of immune checkpoint molecules in high-risk group and low-risk group in luminal breast cancer, HR-Her2+ breast cancer or TNBC. (*p < 0.05, **p < 0.01, and ***p < 0.001).

Then we explored the function of the ferroptosis-related lncRNAs signature in different immunohistochemical subtypes of breast cancer. As shown in Figure 10A, the risk scores based on our ferroptosis-related lncRNAs signature were associated with the overall survival of patients with luminal breast cancer or triple negative breast cancer, and patients with high-risk score had worse prognosis than those with low-risk score. However, due to the small number of patients with HR-Her2+ breast cancer (*n* = 36), the correlation between the risk score and overall survival was not statistically significant in HR-Her2+ breast cancer. And we further investigated the correlations between risk score and tumor immune microenvironment in different immunohistochemical subtypes of breast cancer. In the analysis of luminal breast cancer, the proportions of tumor-infiltrating CD8^+^ T cell, activated NK cell in the high-risk group were significantly lower than those in the low-risk group, while the proportion of tumor-infiltrating M2 macrophages in the high-risk group was much higher than that in the low-risk group (Figure 10B), and the expression of immune checkpoint molecules such as PD1, PDL1, and CTLA4 in the low-risk group were all higher than those in the high-risk group (Figure 10E). Similar results were found in the analysis of triple-negative breast cancer (TNBC) (Figures 10D,G). And we could also find the similar tendency in the analysis of HR-Her2-breast cancer (Figures 10C,F), however, the correlation between risk score and tumor immune microenvironment was not statistically significant due to the small number of patients with HR-Her2+ breast cancer (*n* = 36). These results demonstrated that the ferroptosis-related lncRNAs signature was consistent in different immunohistochemical subtypes of breast cancer.

## Discussion

In this study, we comprehensively studied the relationship between lncRNAs and ferroptosis-related genes in breast cancer, and screened out ferroptosis-related lncRNAs. The expression of ferroptosis-related lncRNAs in breast cancerand their relationship with the prognosis of patients were analyzed, and a novel prognostic model containing 8 ferroptosis-related lncRNAs was constructed, which had a better predictive ability than the AJCC stage. Then we divided the patients into high-risk and low-risk groups based on this prognostic model, and compared the differentially expressed genes between the high-risk group and the low-risk group. Functional enrichment analysis with the differentially expressed genes showed that immune-related pathways were significantly different between the two groups. Finally, we analyzed the infiltration of different immune cells in tumors, and found that breast cancer in the high-risk group was immunologically “cold,” whereas breast cancer in the low-risk group was immunologically “hot”.

Some recent studies have found that lncRNAs play an important role in the ferroptosis of cancers. For example, Wang et al. discovered that long noncoding RNA LINC00336 could inhibit ferroptosis in lung cancer (Wang et al., 2019a). Wang et al. reported that LINC00618 promoted ferroptosis in human leukemia (Wang et al., 2020). However, the number of ferroptosis-related lncRNAs discovered so far is very small. Studies on ferroptosis-related lncRNAs in breast cancer are extremely limited. On the contrary, there have been many reports about genes associated with ferroptosis, so Pearson correlation analysis was performed between discovered ferroptosis-related genes and lncRNAs to identify ferroptosis-related lncRNAs. We identified 11 ferroptosis-related lncRNAs which were significantly correlated with OS of breast cancer patients. Furthermore, eight ferroptosis-related lncRNAs (AC072039.2, LINC01235, TDRKH−AS1, AC108474.1, AL133467.1, LIPE-AS1, MAPT-AS1, and USP30−AS1) were selected to construct a prognostic signature based on their performance in the multivariate Cox regression analysis. LncRNA USP30−AS1 had co-expression relationship with 9 ferroptosis-related genes (CYBB, SOCS1, IFNG, TNFAIP3, PML, GCH1, NCF2, SLC2A6, and CAPG), including 4 ferroptosis driver, 2 ferroptosis suppressor and 3 ferroptosis marker, and upregulation of USP30−AS1 in breast cancer tissue was associated with longer overall survival time in this study. LncRNA LIPE-AS1 was co-expressed with 5 ferroptosis-related genes (HRAS, PHKG2, MAPK14, EGLN2, and GPX4), including 4 ferroptosis driver and 1 ferroptosis suppressor, and we found that LIPE-AS1 was a protective factor for breast cancer patients’ prognosis. And there was a co-expression relationship between AC108474.1 and 5 ferroptosis-related genes (TAZ, ISCU, CAV1, PLIN4, and HIC1), including 1 ferroptosis driver, 2 ferroptosis suppressor and 2 ferroptosis marker, and AC108474.1 was a protective factor for breast cancer patients too.

Ferroptosis is a novel type of cell death characterized by the iron-dependent accumulation of reactive oxygen species and lipid peroxidation (Cao and Dixon, 2016). During the progression of cancers, cancer cells often resist ferroptosis to promote its survival and metastasis (Lu et al., 2017). Some of the drugs used to treat breast cancer, such as anthracycline, cyclophosphamide, paclitaxel and tamoxifen, could induce the production of excess ROS in cancer cells which could cause cell death (Stêrba et al., 2013; Roy et al., 2014; Jiang et al., 2019; Vernier et al., 2020). In theory, ferroptosis-resistant cancer cells would be less sensitive to these drugs, and this is consistent with our findings that high-risk patients had worse outcomes among patients treated with anthracycline, cyclophosphamide, paclitaxel or tamoxifen. But Friedmann Angeli et al. reported that ferroptosis might lead to an immunosuppressive microenvironment to promote cancer progression (Friedmann Angeli et al., 2019). However, the role of ferroptosis, especially ferroptosis-related lncRNAs, in breast cancer immune microenvironment is still unclear. We found that some antioxidant pathways were upregulated in the high-risk group, such as cysteine and methionine metabolism and terpenoid backbone biosynthesis. Cysteine is an important material for the synthesis of glutathione. Increased cysteine can promote the synthesis of glutathione and enhance the antioxidant capacity of breast cancer cells (Wu et al., 2004). Terpenoid is a class of compounds with powerful antioxidant effects, which can resist oxidative damage in cells (González-Burgos and Gómez-Serranillos, 2012). With the up-regulation of antioxidant pathways, oxidative damage-related pathways were significantly down-regulated in the high-risk group, such as GTPases activate NADPH oxidases and oxidative damage. Some studies have found that the activation of NADPH oxidases can promote the production of reactive oxygen species, which causes a series of pathophysiological changes (Roy et al., 2015). So we could reasonably infer that the ferroptosis of cancer cells in high-risk group was inhibited. In addition, we also found significant downregulation of immune-related pathways in the high-risk group, such as antigen processing and presentation, natural killer cell-mediated cytotoxicity, T cell receptor signaling. Therefore, we can reasonably assume that ferroptosis is closely related to anti-tumor immunity in breast cancer. Recent research found that signals released by ferroptotic cells could recruit antigen presenting cells to the site of ferroptosis occurring (Friedmann Angeli et al., 2019). Wang et al. also discovered that CD8^+^ T cells could play an anti-tumor function by promoting tumor ferroptosis (Wang et al., 2019b). These results suggest that ferroptosis is closely related to anti-tumor immunity, which is in accordance with our assumption.

However, the relationship between ferroptosis and immune cell infiltration in breast cancer remains unknown. In our study, CIBERSORT algorithm was used to calculate the proportion of different types of tumor-infiltrating immune cells. The result showed that compared with the low-risk group, the infiltrated tumor-killing immune cells in the breast cancer tissues of the high-risk group were significantly reduced, such as CD8^+^ T cells, γδT cells and activated NK cells, whereas the immune cells promoting tumor proliferation and metastasis, M2 macrophages, were increased (Henning et al., 2018; Hodgins et al., 2019; Sebestyen et al., 2020) (Tariq et al., 2017). Therefore, we can conclude that ferroptosis is significantly correlated with the proportion of tumor-infiltrating immune cells in breast cancer, and breast cancer in high-risk group tended to have less cytotoxic lymphocyte infiltration. Furthermore, breast cancer in high-risk group based on ferroptosis-related-lncRNAs signature tended to have lower expression level of immune checkpoint molecules. Therefore, breast cancer in high-risk group tended to be immunologically “cold,” which was difficult to benefit from immune checkpoint inhibitors, while the low-risk breast cancer based on ferroptosis-related-lncRNAs signature tended to be immunologically “hot,” which was more likely to benefit from immune checkpoint inhibitors (Chen et al., 2017), suggesting that our ferroptosis-related-lncRNAs signature had a potential to predict the efficacy of immune checkpoint blockade therapy for breast cancer patients.

In order to improve the prognosis of high-risk patients, we propose a combined regimen of ferroptosis inducers and immune checkpoint inhibitors to treat breast cancer patients in the high-risk group, which might recruit immune cells by promoting ferroptosis of cancer cells. Then the “cold” tumor could turn into a “hot” tumor, which is sensitive to immune checkpoint inhibitors. This might be a useful treatment regimen. Currently, it has been found that Siramesine and Lapatinib could induce ferroptosis in breast cancer (Ma et al., 2016). Further studies are needed to verify the effect of this combined regimen of ferroptosis inducers and immune checkpoint inhibitors for breast cancer.

There are some limitations in our study. Firstly, our study is based on the TCGA public database, this ferroptosis-related-lncRNAs prognostic model needs to be further verified with prospective, multicenter, real-world data. Secondly, our study only preliminarily revealed the relationship between ferroptosis-related-lncRNAs and anti-tumor immunity. The underlying mechanisms need to be further explored by experiment. Thirdly, the function of ferroptosis-inducing drugs to turn “cold” tumor into “hot” tumor needs to be confirmed by further research.

## Conclusion

In summary, we identified a novel ferroptosis-related lncRNA signature which could precisely predict the prognosis of breast cancer patients. Ferroptosis-related lncRNAs may have a potential role in the process of anti-tumor immunity and serve as therapeutic targets for breast cancer.

## Data Availability

Publicly available datasets were analyzed in this study. This data can be found here: The data of this study were downloaded from TCGA database (https://portal.gdc.cancer.gov/repository).

## Abbreviations

AIC, Akaike information criterion; AUC, Area under time-dependent ROC curve; BC, Breast cancer; BH, Benjamini and Hochberg; FDR, False discovery rate; GSEA, Gene set enrichment analysis; GO, Gene Ontology; ICB, Immune checkpoint blockade; KEGG, Kyoto Encyclopedia of Genes and Genomes; LncRNA, Long non-coding RNA; OS, Overall survival; ROC, Receiver operating characteristic curve; ROS, Reactive oxygen species; TNBC, Triple-negative breast cancer.
