# Supplementary figures and images for: A Ferroptosis-Related lncRNAs Signature Predicts Prognosis and Immune Microenvironment for Breast Cancer
Source: Front Mol Biosci. 2021 Jun 7;8:678877. doi: 10.3389/fmolb.2021.678877 (PMC8215711; doi:10.3389/fmolb.2021.678877)

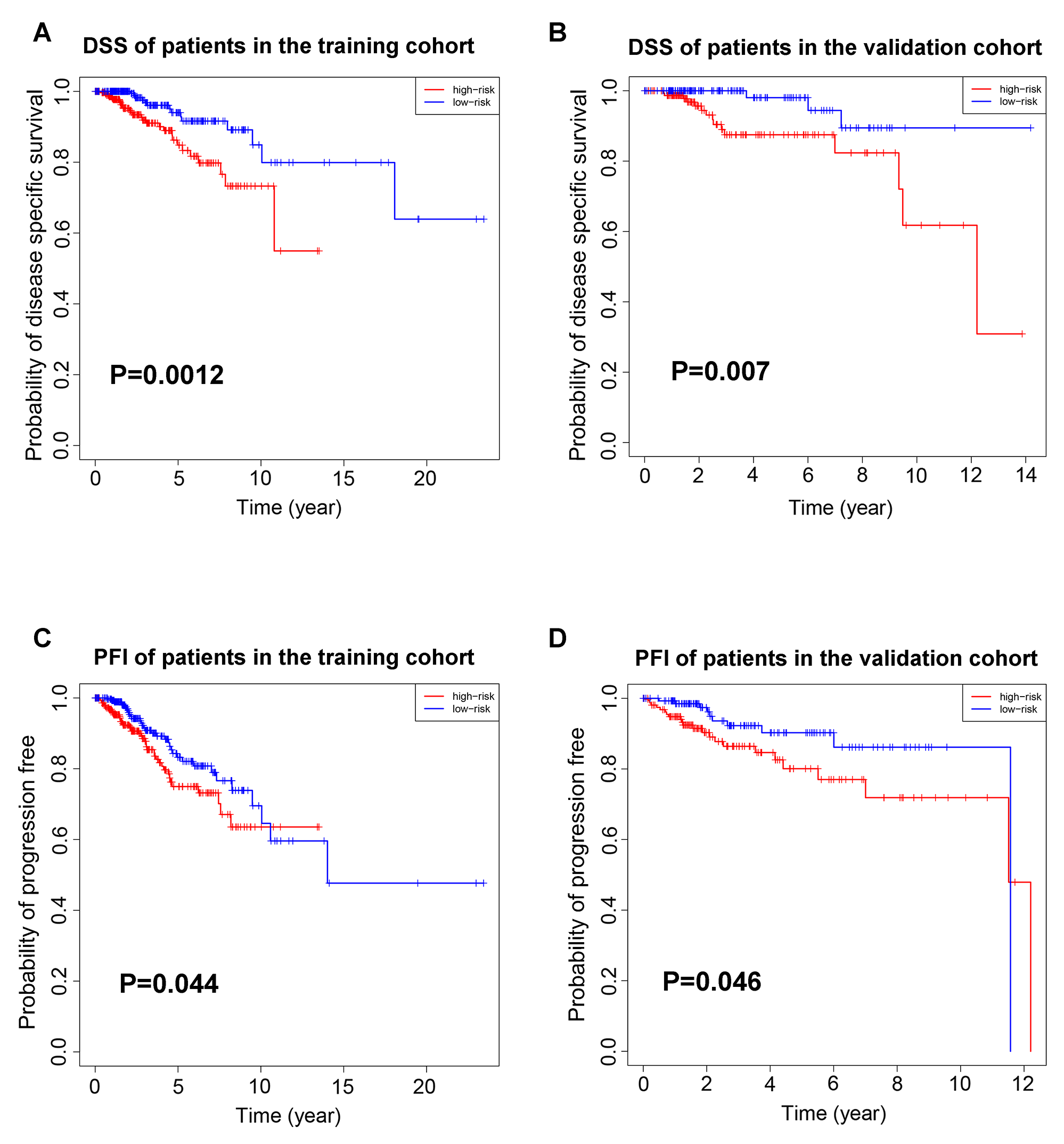

Supplement: Supplementary file 1 [file Image1.TIF]
